# Cor Triatriatum: Case Report of Emergency Department Diagnosis

**DOI:** 10.5811/cpcem.2018.5.37921

**Published:** 2018-06-12

**Authors:** Camille Halfman, Asalim Thabet, Rebecca Blue, Tyler Greenfield

**Affiliations:** SUNY Upstate Medical University, Department of Emergency Medicine, Syracuse, New York

## Abstract

Cor triatriatum is a rare, congenital heart defect. When diagnosis does not occur in infancy, primary symptoms in an older patient may mimic reactive airway disease. We report a case of cor triatriatum in an older child, previously diagnosed with asthma, presenting to an emergency department with a chief complaint of wheezing. Initial treatment with bronchodilators and corticosteroids was unsuccessful, prompting thorough evaluation. Subsequent imaging diagnosed cor triatriatum sinister. When presentations consistent with common conditions, such as asthma, do not respond appropriately to classic intervention, emergency physicians must be prepared to consider alternative and rare diagnosis.

## INTRODUCTION

**Image 1:**
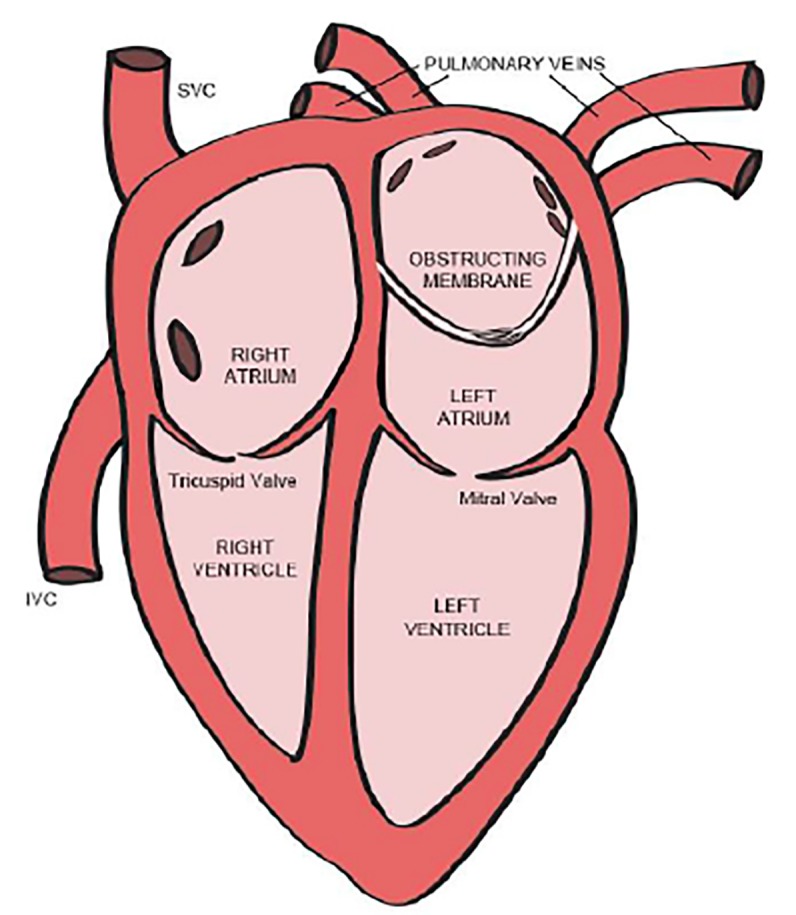
Cor triatriatum sinister. Classic findings include a membrane separating the left atrium into two components: one chamber with the pulmonary veins and the other with the mitral valve and atrial appendage. The obstructing membrane is often partially fenestrated, allowing communication between the proximal and distal atrial segments. *SVC*, superior vena cava; *IVC*, inferior vena cava.

Cor triatriatum is one of the rarest forms of congenital heart defect, estimated incidence of 0.1% of all the congenital heart diseases.^1^ Classically, there is a membrane that separates the left atrium into two components: one chamber with the pulmonary veins and the other with the mitral valve and atrial appendage (Image 1). Most patients are identified shortly after birth with the evaluation of a distressed or cyanotic neonate. However, when presentation is delayed, primary symptoms may mimic reactive airway disease.^2^ We report a case of cor triatriatum in an older child who presented to an emergency department (ED) with a chief complaint of wheeze and dyspnea with a previous diagnosis of asthma.

## CASE REPORT

A five-year-old male with reported history of poor weight gain and mild intermittent “asthma” presented to the pediatric ED in respiratory distress. He was tachypneic and tachycardic, with an oxygen saturation of 86% on room air. According to the patient’s mother, he had been seen by a pediatric pulmonologist approximately two months prior and found to have normal pulmonary function tests that did not change with albuterol administration. He was diagnosed with asthma and given prescriptions for budesonide/formoterol and albuterol nebulizer.

The patient had further presented to his primary pediatrician approximately one month before his ED visit for complaints of fever. At that time he was diagnosed with acute otitis media and started on azithromycin, but returned four days later with increasing wheezing, upper respiratory symptoms, and exercise intolerance. His antibiotic was changed to cefdinir with a five-day course of prednisolone; the mother reported that he improved with this regimen. He had otherwise been in “normal” health since that time, though his mother did endorse continued issues of poor weight gain and intermittent wheezing.

On the day of presentation, the mother reported that the patient was unable to tolerate a single flight of stairs without fatigue and wheezing. He had used the budesonide/formoterol inhaler earlier that morning and had received multiple albuterol nebulizer treatments prior to arrival without improvement. The patient’s respiratory status had been worsening for the previous two days, with increased dyspnea and wheezing on exertion the day prior to arrival such that he could not play for more than 10 minutes outside without becoming severely dyspneic and fatigued.

On initial exam, the patient was notably tachypneic and tachycardic as well as hypoxic on room air. An expiratory wheeze was appreciated, but no obvious murmur was heard on cardiac auscultation. Splenomegaly was noted. Given the reported history, nebulized albuterol with ipratropium was ordered. Following the breathing treatment, auscultation demonstrated improvement of wheezes, though bilateral coarse breath sounds were appreciated at that time. He remained tachypneic and tachycardic but improved to the point that he was able to speak in full sentences. Oxygen saturation initially improved to 95% but quickly declined to 85% when the nebulizer treatment was completed. Work of breathing increased drastically with worsening hypoxia.

**Image 2:**
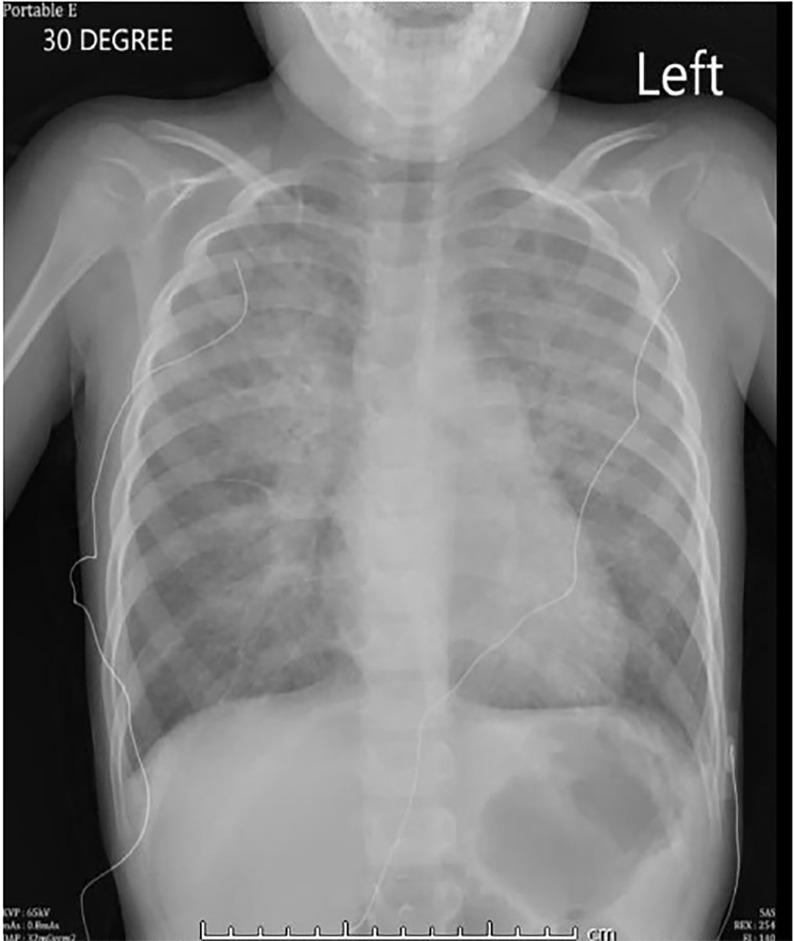
Initial chest radiograph demonstrating pulmonary edema.

**Image 3:**
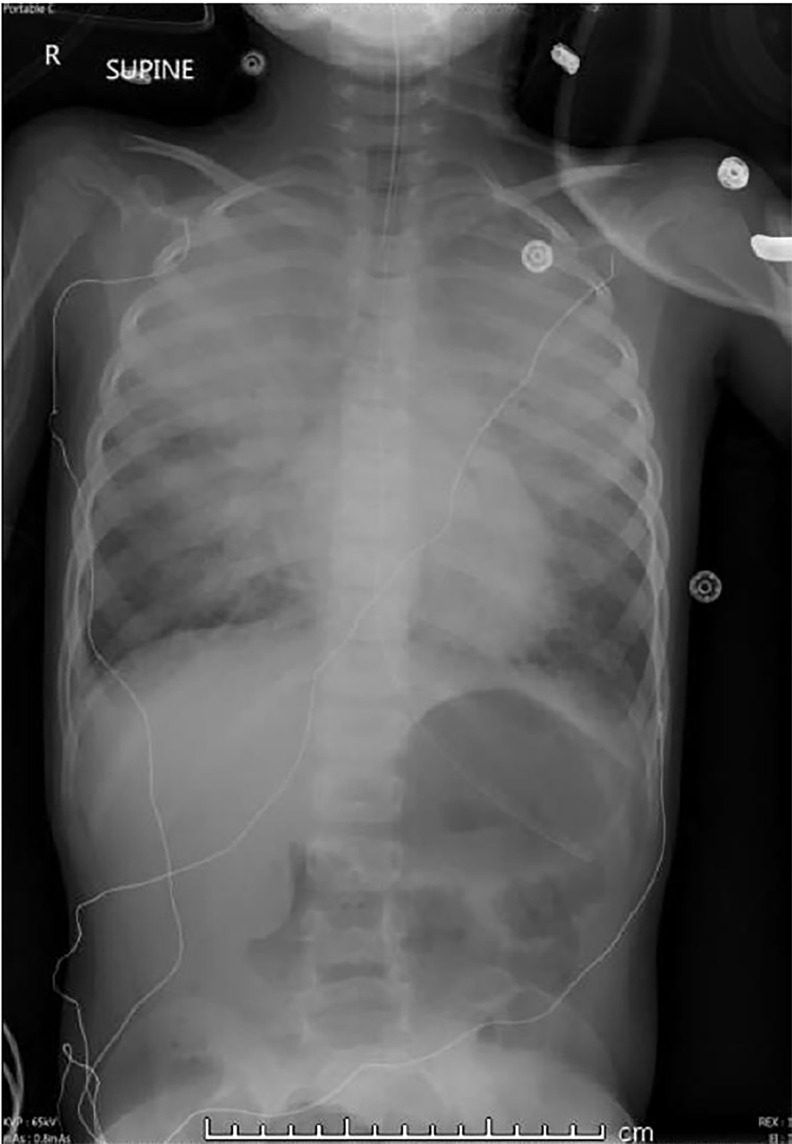
Post-Intubation chest radiograph demonstrating worsening edema consistent with acute respiratory distress syndrome.

The patient was started on continuous albuterol and given magnesium, solumedrol, and ceftriaxone without significant improvement. Chest radiography demonstrated severe pulmonary edema (Image 2). At this time, the patient’s respiratory and mental status statuses rapidly declined and intubation was indicated. He was successfully intubated, but confirmatory chest radiograph demonstrated worsening edema consistent with acute respiratory distress syndrome (Image 3). At this time the patient was admitted and care was transferred to the pediatric intensive care unit (PICU).

After transfer to the PICU, the patient’s oxygen saturation gradually improved to 95% on 100% fraction of inspired oxygen (FiO_2_) with a high positive end-expiratory pressure. The pediatric cardiology team was consulted and performed a bedside echocardiogram that revealed cor triatriatum with severe supravalvar mitral stenosis and significant pulmonary hypertension. The patient was transferred for surgical correction of the malformation as pediatric cardiac surgery was not available at the admitting institution.

**CPC-EM Capsule**

What do we already know about this clinical entity?

Cor triatriatum is one of the rarest forms of congenital heart defect, estimated incidence of 0.1% of all the congenital heart disease.

What makes this presentation of disease reportable?

While most cases of cor triatriatum are diagnosed in early infancy, rare cases can present with primarily pulmonary complaints, leading to delayed or missed diagnostic opportunities.

What is the major learning point?

Pulmonary complaints that do not respond appropriately to common therapeutic interventions should raise emergency provider suspicion for alternative disease processes.

How might this improve emergency medicine practice?

This case highlights the need for diagnostic acumen and the expansion of differential and appropriate imaging in a case where a common presentation does not translate to a common diagnosis.

## DISCUSSION

Cor triatriatrum sinister is an exceedingly rare congenital cardiac malformation characterized by the presence of a membrane separating the left atrium into two components: one chamber with the pulmonary veins and the other with the mitral valve and atrial appendage.^3^ A communication between the right atrium and the proximal or distal chamber is not uncommon.^4^,^5^ The membrane arises in relationship to the left superior vena cava (LSVC); it is hypothesized that the LSVC normally obliterates during early embryonic atrial development but a prominent or persistent LSVC may induce an abnormal left atrial membrane by impingement.^6^

While the condition can occur in the right heart (cor triatriatum dexter), it is more common in the left (sinister).^3^ If there is a remaining communication between the divided atrial chambers of sufficient size to allow circulatory exchange, clinical presentation can be delayed from infancy until later childhood or even adulthood.^3^,^7^ Such presentation may closely mimic signs and symptoms of left heart outflow obstruction. Children may exhibit signs of poor weight gain and failure to thrive, potentially leading to delayed motor milestones.^1^,^8^ In addition to respiratory complaints, adolescents may complain of gastrointestinal symptoms including abdominal pain, nausea, and weight loss.^8^ Symptomatic atrial dysrhythmias may be seen.^1^

Misdiagnosis of cor triatriatum as reactive airway disease or asthma has been reported occasionally.^2^,^3^,^9^,^10^ Even so, the diagnosis remains elusive and challenging particularly in the ED. Given the prevalence of asthma and other reactive airway disorders in childhood, with up to 11% of all children aged 10 years having episodes of reactive airway disease,^11^ it is unsurprising that a presentation as in the case above would prompt initial ED management with bronchodilators, inhaled beta-agonists, and corticosteroids. However, poor response to such treatment mainstays or unexpectedly poor pulmonary function should prompt imaging and consideration of less-common causes of wheeze. Definitive diagnosis of cor triatriatum is easily made by echocardiogram.

Previous publications regarding the association between cor triatriatum and wheezing have suggested that the etiology may be related primarily to pulmonary hypertension, with pulmonary venous hypertrophy triggering spasm of pulmonary vasculature and associated bronchial smooth muscle reactivity.^2^,^3^ Initial improvement with bronchodilators may be observed given that the final mechanism remains smooth muscle activation and resultant airway narrowing. As in this case, such response would be expected to be short-lived, with the patient experiencing additional pulmonary decline from pulmonary edema and associated respiratory distress. Temporizing management of acute pulmonary edema with non-invasive or invasive ventilatory support, or through cautious preload/afterload reduction or pulmonary vasodilation, may be helpful in stabilization prior to definitive intervention. Surgical correction offers good results once the correct diagnosis is made.^1^

## CONCLUSION

Common presentations in the ED prompt common interventions; most pediatric presentations of wheezing are indeed associated with reactive airways. However, emergency providers should not rely on the common diagnoses when faced with poor response to seemingly appropriate interventions. While congenital cardiac malformations are most commonly diagnosed early in childhood, undiagnosed cases may present as clinical quandaries that should prompt more thorough evaluation. Cor triatriatum is rare, particularly in older children, but can be diagnosed by the discerning emergency physician through careful examination, evaluation of treatment response, and expanded imaging when a patient does not appropriately respond to common therapeutic interventions. Whether or not definitive diagnosis is made, identification of pulmonary edema, regardless of etiology, should prompt appropriate management of oxygenation and ventilation, medical stabilization, and consultation for further care.

Documented patient informed consent and/or Institutional Review Board approval has been obtained and filed for publication of this case report.
